# Supplementary material for: Sympathetic innervation of interscapular brown adipose tissue is not a predominant mediator of Oxytocin (OT)-elicited reductions of body weight gain and adiposity in male diet-induced obese rats
Source: Front Drug Deliv. 2024 Dec 3;4:1497746. doi: 10.3389/fddev.2024.1497746 (PMC11759500; doi:10.3389/fddev.2024.1497746)
Supplement: Supplementary file 4 [file DataSheet1.docx]

#### Supplemental Study 1: Determine if surgical denervation of IBAT blocked the ability of 4V OT to increase T_IBAT_ in lean rats. Rats (N= 6 at study onset) were used for this study. The goal of this study was to determine if OT- elicited increase in T_IBAT_ requires intact SNS outflow to IBAT in lean rats. By design, rats were lean as determined by both BW (511.6 ± 2.4 g) and adiposity (3.3 ± 0.3 g fat mass; 18.1 ± 11.1% adiposity) after maintenance on the chow (13% kcal from fat; N=6/group) for approximately 1 week prior to denervation procedures and implantation of temperature transponders underneath IBAT. Rats were otherwise treated identically to those used in Study 4.

**Supplemental Study 1:**

Tissue samples from **Supplemental Study 1** were screened for NE content and compared with a previous cohort of sham-operated rats from **Study 1**. However, there was no age-matched cohort to use for comparison purposes to calculate reduction of IBAT NE content as the cohort used in **Supplemental Study 1** was heavier than the sham-operated cohort used in **Study 1** (354 ± 4.6 g) [(F(1,9) = 608.553, *P*<0.001]. IBAT NE content from sham-operated animals was only used to screen whether IBAT NE content was above the acceptable threshold for a successful IBAT denervation. One of the six animals was removed on account of having a failed IBAT denervation procedure NE content was reduced by 93.8 ±2.6% in IBAT from denervated rats relative to IBAT from sham-operated control rats [(F(1,9) = 16.390, *P*<0.01]. IWAT NE content was also reduced in the denervated group [(F(1,9) = 7.411, *P*<0.05)] but this might have been due, in part, to the differences in age of the animals between cohorts. In contrast, NE content was unchanged in pancreas but elevated in liver [(F(1,9) = 8.684, *P*<0.05] in denervated rats relative to sham rats.

In denervated rats, 4V OT (5 μg/μL) increased T_IBAT_ at 0.5, 0.75, 1, 1.25, 1.5 and 1.75-h post-injection (*P*<0.05; **Supplemental Figure 1A**). The high dose (5 μg/μL) also stimulated T_IBAT_ when measuring change in T_IBAT_ relative to baseline T_IBAT_ at 0.5, 0.75, 1, 1.25, 1.5, 1.75 and 2-h post-injection (*P*<0.05; **Supplemental Figure 1B**). The low dose (1 μg/μL) increased T_IBAT_ at 1.5-h post-injection (*P*<0.05; **Supplemental Figure 1A**). The low dose (1 μg/μL) produced a near significant stimulation of T_IBAT_ when measuring change in T_IBAT_ relative to baseline T_IBAT_ at 1- and 1.5-h post-injection (0.05<*P*<0.1; **Supplemental Figure 1B**).

**Supplemental Figure 1A–B: Effect of 4V OT on IBAT temperature (T_IBAT_) in lean rats.** Rats (N=6 total) were fed *ad libitum* and maintained on low fat chow diet (13% kcal from fat) (N=6/group) for approximately 1 week prior to underdoing SNS denervation procedures and implantation of temperature transponders underneath the left IBAT depot. Rats subsequently received 4V cannulations. Rats were allowed to recover for at least 2 weeks during which time they were adapted to a daily 4-h fast, handling, and mock injections. Rats subsequently received injections of 4V OT (1 or 5 μg/μL) or vehicle where each animal received each treatment at least 48-h intervals. *A*, Effect of 4V OT on T_IBAT_ in IBAT denervated lean rats; *B*, Effect of 4V OT on change in T_IBAT_ relative to baseline T_IBAT_ (delta T_IBAT_) in IBAT denervated lean rats. Data are expressed as mean ± SEM. **P*<0.05, †0.05<*P*<0.1 4V OT vs. vehicle.

**Supplemental Study 2: Determine the extent to which β3-AR-induced activation of IBAT requires activation of β3-AR to increase T_IBAT_ in DIO rats.**

Rats (N= 19 at study onset) were used for this study. Rats were fed *ad libitum* and maintained on HFD for approximately 4.5 months prior to receiving 4V cannulas and/or temperature transponders [[28](#_ENREF_28)]. Rats were allowed to recover for at least 4 weeks during which time they were adapted to a daily 4-h fast, handling, and mock injections.

The goal of this study was to determine a dose of the β3-AR antagonist that was sufficient to block the effects of the β3-AR agonist on T_IBAT_ in DIO rats. The dose of the β3-AR antagonist would then be used in **Study 6** to determine if 4V OT required β3-AR signaling to stimulate T_IBAT_ in DIO rats. By design, DIO rats were obese as determined by both BW (712 ± 23.1 g) and adiposity (217 ± 12.1 g fat mass; 31 ± 1.0% adiposity) after maintenance on the HFD for 4.5 months prior to sham/denervation procedures.

In the absence of the β3-AR antagonist, the β3-AR agonist increased T_IBAT_ at 0.25, 0.5, 0.75, 1, 1.5, 0.75, 2 and 4-h post-injection (*P*<0.05) and produced a near significant stimulation of T_IBAT_ at 180-min post-injection (0.05<*P*<0.1) (**Supplemental Figure 2**). These effects were blocked at 0.5-h post-injection of the β3-AR agonist. Together, these findings confirm the specificity of a β3-AR mediated effect of the β3-AR agonist on T_IBAT_ and identified a dose of the β3-AR antagonist that was sufficient to block the effects of the β3-AR agonist to stimulate T_IBAT_.

Two-way repeated-measures ANOVA revealed a signiﬁcant main effect of β3-AR agonist to increase T_IBAT_ at 0.5-h post-injection [F(1,48) = 5.844, *P*=0.019], a significant main effect of the β3-AR antagonist on T_IBAT_ at 0.5-h post-injection [F(1,48) = 5.671, *P*=0.021] and a signiﬁcant interaction between the β3-AR agonist and the β3-AR antagonist on T_IBAT_ at 0.5-h post-injection [F(1,48) = 5.728, *P*=0.021].

Overall, these findings confirmed the specificity of a β3-AR mediated effect of the β3-AR agonist on T_IBAT_ and identified a dose of the β3-AR antagonist that was sufficient to block the effects of the β3-AR agonist to stimulate T_IBAT_.

**Supplemental Figure 2A–B: Effect of β3-AR antagonist, SR59230A, on the ability of the β3-AR agonist, CL 316243, to increase T_IBAT_ in DIO rats.** Rats (N=17 total) were maintained on HFD (60% kcal from fat; N=17/group) for approximately 4.25 months prior to being implanted with temperature transponders underneath IBAT. Animals were subsequently adapted to a 4-h fast prior to receiving acute IP injections of the β3-AR agonist, SR59230A, or vehicle approximately 20 minutes prior to IP injections of the β3-AR agonist, CL 316243. *A*, Effect of the β3-AR antagonist (SR-59230A) pre-treatment on the ability of the β3-AR agonist, CL 316243, to stimulate T_IBAT_ at 0.5-h post-injection; *B*, Effect of the β3-AR antagonist (SR-59230A) pre-treatment on the ability of the β3-AR agonist, CL 316243, to stimulate T_IBAT_ at 0.75-h post-injection. Data are expressed as mean ± SEM. **P*<0.05, †0.05<*P*<0.1 OT vs. vehicle.

**Supplemental Study 3: Determine the extent to which OT-induced activation of sympathetic outflow to IBAT contributes to its ability to elicit weight loss in rats with more pronounced diet-induced obesity.**

Rats (N= 20 at study onset) were used for this study. Rats were fed *ad libitum* and maintained on HFD for approximately 8.75 months prior to receiving 4V cannulas and minipumps to infuse vehicle or OT (16 nmol/day) over 29 days as previously described [[28](#_ENREF_28)]. Daily energy intake and BW were also tracked for 29 days. Animals were euthanized by rapid conscious decapitation at 9 weeks post-sham or denervation procedure. Trunk blood and tissues (IBAT, EWAT, IWAT, liver and pancreas) were collected from 4-h fasted rats and tissues were subsequently analyzed for IBAT NE content to confirm success of denervation procedure relative to sham operated animals and other tissues (EWAT, IWAT, liver and pancreas).

The goal of this study was to determine if OT-elicited weight loss requires intact SNS outflow to IBAT. By design, DIO rats were obese as determined by both BW (790.7 ± 23.8 g) and adiposity (281.9 ± 16.3 g fat mass; 35.3 ± 0.1.3% adiposity) after maintenance on the HFD for 8.75 months prior to sham/denervation procedures.

All IBAT tissues from **Supplemental Study 3** animals were analyzed for IBAT NE content and one of the ten animals were removed on account of having a failed IBAT denervation procedure IBAT NE content was reduced in denervated rats by 84.8±4.3% in denervated rats relative to sham-operated control rats [(F(1,16) = 128.544, *P*=0.000)]. In contrast, NE content was unchanged in IWAT, EWAT or pancreas in denervated rats relative to sham rats (*P*=NS). In contrast, there was a reduction of NE content in liver in denervated rats relative to sham rats [(F(1,16) = 4.654, *P*=0.047)]. There was no significant difference in BW between sham and denervation groups at the end of the study (*P*=NS; data not shown).

In sham-operated rats, as expected, 4V vehicle resulted in 4.3 ± 1.0% weight gain relative to vehicle pre-treatment [(F(1,4) = 15.268, *P*=0.017]. In contrast, 4V OT reduced BW by 4.2 ± 1.2% relative to OT pre-treatment [(F(1,4) = 10.931, *P*=0.030] (**Supplemental** **Figure 3A**) and it also reduced weight gain throughout the 29-day infusion period (**Supplemental Figure 3B**). OT treatment reduced weight gain on days 4-29 (*P*<0.05) and produced a near significant reduction of weight gain on day 3 (*P*=0.068). 4V OT reduced relative fat mass (pre- vs post-intervention; **Supplemental Figure 3C**) (*P*<0.05) and produced a reduction in the relative lean body mass (pre- vs post-intervention; *P*<0.05) without impacting total fat mass or lean body mass (*P*=NS). These effects that were mediated, at least in part, by a modest reduction of energy intake that persisted throughout the first two weeks of the treatment period (**Supplemental Figure 3D**; *P*<0.05). 4V OT also produced a near significant reduction of energy intake during weeks 3 and 4 of the treatment period (**Supplemental Figure 3D**; 0.05<*P*<0.1).

In denervated rats, as expected, 4V vehicle resulted in 4.7 ± 0.8% weight gain relative to vehicle pre-treatment [(F(1,3) = 44.734, *P*=0.007] (**Supplemental Figure 3A**). In contrast, 4V OT failed to reduce BW relative to OT pre-treatment but it reduced weight gain throughout the 29-day infusion period (**Supplemental** **Figure 3B**). OT treatment reduced weight gain on days 4-29 (*P*<0.05) and it produced a near significant reduction of weight gain on day 3 (*P*=0.05). 4V OT reduced relative fat mass (pre- vs post-intervention; **Supplemental Figure 3C**) (*P*<0.05) without effecting total fat mass or lean body mass (*P*=NS). These effects that were mediated, at least in part, by a modest reduction of energy intake that was evident during weeks 1 and 2 of the treatment period (**Supplemental** **Figure 3D**; *P*<0.05).

There was no significant difference in BW between sham and denervation groups at the end of the study (≈ 9-weeks post-sham/denervation surgery) (*P*=NS; data not shown).

Two-way ANOVA revealed a signiﬁcant main effect of OT to reduce body weight gain on day 29 [F(1,14) = 39.841, *P*<0.01] but no overall effect of denervation [F(1,14) = 0.292, *P*=0.598] or an interactive effect between OT and denervation on body weight gain on day 29 [F(1,14) = 0.094, *P*=0.908]. Two-way ANOVA revealed no significant main effect of OT [F(1,14) = 1.278, *P*=0.277] or denervation on fat mass [F(1,14) = 0.051, *P*=0.825] or an interactive effect between OT and denervation on fat mass [F(1,14) = 0.443, *P*=0.517].

Overall, these ﬁndings demonstrate that sympathetic innervation of IBAT is not a predominant mediator of 4V OT-elicited reductions of BW and adiposity in male DIO rats.

**Supplemental Figure 3A–D: Effect of chronic 4V OT infusions (16 nmol/day) on BW, adiposity, and energy intake post-sham or IBAT denervation in male DIO rats with more pronounced diet-induced obesity.** *A*, Rats (N=18 total) were maintained on HFD (60% kcal from fat; N=4-5/group) for approximately 8.75 months prior to undergoing a sham or bilateral surgical IBAT denervation. Rats were subsequently implanted with 4V cannulas and allowed to recover for 2 weeks prior to being implanted with subcutaneous minipumps that were subsequently attached to the 4V cannula. *A*, Effect of chronic 4V OT or vehicle on BW in sham operated or IBAT denervated DIO rats; *B*, Effect of chronic 4V OT or vehicle on BW change in sham operated or IBAT denervated DIO rats; *C*, Effect of chronic 4V OT or vehicle on adiposity in sham operated or IBAT denervated DIO rats; *D*, Effect of chronic 4V OT or vehicle on adiposity in sham operated or IBAT denervated DIO rats. Data are expressed as mean ± SEM. **P*<0.05, †0.05<*P*<0.1 OT vs. vehicle.
